# Routine 36‐week scan: prediction of small‐for‐gestational‐age neonate

**DOI:** 10.1002/uog.29134

**Published:** 2024-11-25

**Authors:** S. Adjahou, A. Syngelaki, M. Nanda, D. Papavasileiou, R. Akolekar, K. H. Nicolaides

**Affiliations:** ^1^ Fetal Medicine Research Institute King's College Hospital London UK; ^2^ Fetal Medicine Unit Medway Maritime Hospital Gillingham UK; ^3^ Institute of Medical Sciences Canterbury Christ Church University Chatham UK

**Keywords:** adverse perinatal outcome, estimated fetal weight, fetal biometry, pyramid of pregnancy care, small‐for‐gestational age, symphysis–fundus height, third‐trimester screening

## Abstract

**Objectives:**

First, to compare the predictive performance of routine ultrasonographic estimated fetal weight (EFW) at 31 + 0 to 33 + 6 and 35 + 0 to 36 + 6 weeks' gestation for delivery of a small‐for‐gestational‐age (SGA) neonate. Second, to compare the predictive performance of EFW at 36 weeks' gestation for SGA *vs* fetal growth restriction (FGR) at birth. Third, to compare the predictive performance for delivery of a SGA neonate of EFW < 10^th^ percentile *vs* a model combining maternal demographic characteristics and elements of medical history with EFW.

**Methods:**

This was a retrospective analysis of prospectively collected data in 21 676 women with a singleton pregnancy who had undergone routine ultrasound examination at 31 + 0 to 33 + 6 weeks' gestation and 107 875 women with a singleton pregnancy who had undergone routine ultrasound examination at 35 + 0 to 36 + 6 weeks. Measurements of fetal head circumference, abdominal circumference and femur length were used to calculate EFW according to the Hadlock formula and this was expressed as a percentile according to the Fetal Medicine Foundation fetal and neonatal population weight charts. The same charts were used to diagnose SGA neonates with birth weight < 10^th^ or < 3^rd^ percentile. FGR was defined as birth weight < 10^th^ percentile in addition to Doppler anomalies. For each gestational‐age window at screening, the screen‐positive rate and detection rate were calculated at different EFW cut‐offs between the 10^th^ and 50^th^ percentiles for predicting the delivery of a SGA neonate with birth weight < 10^th^ or < 3^rd^ percentile, either within 2 weeks or at any time after assessment. The areas under the receiver‐operating‐characteristics curves (AUC) of screening for a SGA neonate by EFW at 31 + 0 to 33 + 6 weeks and at 35 + 0 to 36 + 6 weeks were compared.

**Results:**

The predictive performance of routine ultrasonographic examination during the third trimester for delivery of a SGA neonate is higher if: first, the scan is carried out at 35 + 0 to 36 + 6 weeks' gestation rather than at 31 + 0 to 33 + 6 weeks; second, the outcome measure is birth weight < 3^rd^ rather than < 10^th^ percentile; third, the outcome measure is FGR rather than SGA; fourth, if delivery occurs within 2 weeks after assessment rather than at any time after assessment; and fifth, prediction is performed using a model that combines maternal demographic characteristics and elements of medical history with EFW rather than EFW < 10^th^ percentile alone. At 35 + 0 to 36 + 6 weeks' gestation, detection of ≥ 85% of SGA neonates with birth weight < 10^th^ percentile born at any time after assessment necessitates the use of EFW < 40^th^ percentile. Screening at this percentile cut‐off predicted 95% and 98% of neonates with birth weight < 10^th^ and < 3^rd^ percentile, respectively, born within 2 weeks after assessment, and the respective values for neonates born at any time after assessment were 85% and 93%.

**Conclusion:**

Routine third‐trimester ultrasonographic screening for a SGA neonate performs best when the scan is carried out at 35 + 0 to 36 + 6 weeks' gestation, rather than at 31 + 0 to 33 + 6 weeks, and when EFW is combined with maternal risk factors to estimate the patient‐specific risk. © 2024 The Author(s). *Ultrasound in Obstetrics & Gynecology* published by John Wiley & Sons Ltd on behalf of International Society of Ultrasound in Obstetrics and Gynecology.

## INTRODUCTION

Small‐for‐gestational‐age (SGA) neonates are at increased risk of perinatal mortality and morbidity, but this risk can be reduced substantially if SGA is identified prenatally, because close monitoring, appropriate timing of delivery and prompt neonatal care can be undertaken^1^, ^2^. The traditional approach to identifying pregnancies with a SGA fetus is maternal abdominal palpation and serial measurements of symphysis–fundus height, but the detection rate (DR) of this approach is less than 30%^3^, ^4^. Improved screening for SGA is achieved by sonographic fetal biometry for determination of estimated fetal weight (EFW) during the third trimester. There is evidence that better prediction of SGA neonates is achieved if: first, the method of screening is routine third‐trimester biometry rather than selective ultrasonography based on maternal risk factors and serial measurements of symphysis–fundus height^5^, ^6^; second, the routine ultrasound examination is carried out at 36 weeks rather than at 32 weeks' gestation^7^, ^8^, ^9^, ^10^; and third, EFW is combined with maternal demographic characteristics and elements of medical history (henceforth referred to as maternal factors) to derive patient‐specific risks rather than using EFW < 10^th^ percentile alone^11^, ^12^.

The objectives of this study, which is considerably larger than those published previously by our group, were: first, to compare the predictive performance of routine ultrasonographic EFW at 36 weeks *vs* 32 weeks for the delivery of a SGA neonate; second, to compare the predictive performance of EFW at 36 weeks' gestation for SGA *vs* fetal growth restriction (FGR) at birth; and third, to compare the predictive performance for delivery of a SGA neonate of EFW < 10^th^ percentile *vs* a model combining maternal factors with EFW.

## METHODS

### Study population and design

This was a retrospective analysis of prospectively collected data in women with a singleton pregnancy that had undergone routine ultrasound examination in the third trimester at King's College Hospital, London, UK, or Medway Maritime Hospital, Gillingham, UK, between May 2011 and November 2023. In the participating hospitals, all women with a singleton pregnancy are offered routine ultrasound examinations at 11 + 0 to 13 + 6 and 19 + 0 to 23 + 6 weeks' gestation. During the period between May 2011 and March 2014, an additional scan was offered at 31 + 0 to 33 + 6 weeks (dataset A, *n* = 21 676) but, subsequently, between April 2014 and November 2023, this was changed to 35 + 0 to 36 + 6 weeks (dataset B, *n* = 107 875). The timing of the routine third‐trimester scan was changed because the performance of screening for delivery of a SGA neonate and term pre‐eclampsia at the 32‐week assessment was found to be poor. It was hypothesized that, if the assessment was carried out at 36 weeks, the performance of screening would be higher. During the selection of patients for the two datasets, care was taken to include only routine scans and not follow‐up scans for maternal medical conditions or a suspected fetal growth anomaly.

At the third‐trimester visit, maternal demographic characteristics and medical history were recorded. An ultrasound scan was carried out for examination of fetal anatomy and measurement of fetal head circumference, abdominal circumference and femur length in order to calculate EFW using the formula of Hadlock *et al*.^13^, which was identified in a systematic review as the most accurate model for EFW^14^. Additionally, for the purpose of this study, at the 35 + 0 to 36 + 6‐week scan, we carried out transabdominal color Doppler ultrasound for measurement of mean uterine artery (UtA) pulsatility index (PI), umbilical artery (UA) PI and fetal middle cerebral artery (MCA) PI^15^, ^16^.

Gestational age was determined by the measurement of fetal crown–rump length at 11–14 weeks or fetal head circumference at 19–24 weeks^17^, ^18^. Ultrasound scans were performed by sonographers who had extensive training in ultrasound scanning and had obtained the appropriate Fetal Medicine Foundation (FMF) certificate of competence in ultrasound and Doppler examinations (www.fetalmedicine.com).

The inclusion criteria for the study were singleton pregnancy delivering a non‐malformed liveborn or stillborn baby at ≥ 35 + 0 weeks' gestation. We excluded pregnancies with aneuploidy and those with major fetal abnormality. As this study was a retrospective analysis of data derived from routine clinical examinations, ethics committee approval was not required.

### Outcome measures

Data on pregnancy outcome were collected from the hospital maternity records or the general medical practitioners of the women. The outcome measures were delivery of a neonate with birth weight < 10^th^ or < 3^rd^ percentile, based on the FMF fetal and neonatal population weight charts^19^.

### Statistical analysis

Data are expressed as median (interquartile range) for continuous variables and *n* (%) for categorical variables. The Mann–Whitney *U*‐test and chi‐square test or Fisher's exact test were used for comparing outcome groups for continuous and categorical data, respectively. Significance was assumed at 5%.

The observed measurements of EFW and birth weight were converted to *Z*‐scores and percentiles adjusted for gestational age, according to the FMF fetal and neonatal population weight charts^19^. Logistic regression analysis was undertaken to determine the significance of the contribution of EFW *Z*‐score to the prediction of delivering a SGA neonate with birth weight < 10^th^ or < 3^rd^ percentile. The performance of screening was determined by receiver‐operating‐characteristics (ROC)‐curve analysis, and the areas under the ROC curves (AUC) for screening at 31 + 0 to 33 + 6 weeks and at 35 + 0 to 36 + 6 weeks in the prediction of SGA at birth were compared. For each gestational‐age window, the screen‐positive rate and DR were calculated at different EFW cut‐offs between the 10^th^ and 50^th^ percentiles for predicting the delivery of a SGA neonate with birth weight < 10^th^ or < 3^rd^ percentile, either within 2 weeks after assessment at ≥ 35 + 0 weeks' gestation or at any time after assessment. Similarly, the performance of screening for FGR was determined; the diagnosis of FGR was based on a combination of birth weight < 10^th^ percentile with Doppler findings of UtA‐PI or UA‐PI ≥ 95^th^ percentile or MCA‐PI ≤ 5^th^ percentile.

The maternal factor‐related risk for SGA was derived in the dataset of 107 875 singleton pregnancies seen at 35 + 0 to 36 + 6 weeks' gestation (dataset B) using multivariable logistic regression analysis with backward stepwise elimination to determine which of the factors among maternal demographic characteristics and medical and obstetric history had a significant contribution in predicting the delivery of a SGA neonate with birth weight < 10^th^ percentile. Prior to the regression analysis, the continuous variables, such as age, weight and height, were centered by subtracting the arithmetic mean from each value. Categorical variables were dummy coded as binary or ordinal variables to estimate the independent effect of each category. Multivariable logistic regression analysis was then used to determine if the maternal factor‐derived logit (prior risk) plus EFW *Z*‐score was superior to EFW < 10^th^ percentile in predicting the delivery of a SGA neonate with birth weight < 10^th^ or < 3^rd^ percentile within 2 weeks and at any time after assessment.

The statistical software packages SPSS version 24.0 (IBM Corp., Armonk, NY, USA) and Medcalc (Medcalc Software, Mariakerke, Belgium) were used for statistical analysis.

## RESULTS

### Patient characteristics

The baseline characteristics of the study population (*n* = 129 551) are shown in Table 1. The characteristics of women who underwent an ultrasound scan at 31 + 0 to 33 + 6 weeks' gestation (May 2011 to March 2014) were similar to those of women with a scan at 35 + 0 to 36 + 6 weeks (April 2014 to November 2023). In both study periods, in the group of neonates with birth weight < 10^th^ percentile, compared to those with birth weight ≥ 10^th^ percentile, the median maternal age, weight and height, EFW *Z*‐score, birth‐weight *Z*‐score and gestational age at delivery were lower, the frequency of non‐white ethnicity, chronic hypertension, cigarette smoking and previous pregnancy affected by SGA was higher, and fewer women were parous with no previous SGA.

**Table 1:** Maternal and pregnancy characteristics of study population

	Screening at 31 + 0 to 33 + 6 weeks		Screening at 35 + 0 to 36 + 6 weeks	
Characteristic	BW ≥ 10^th^ percentile (*n* = 19 004)	BW < 10^th^ percentile (*n* = 2672)	BW ≥ 10^th^ percentile (*n* = 95 455)	BW < 10^th^ percentile (*n* = 12 420)
Maternal age (years)	30.7 (26.1–34.5)	29.7 (24.8–34.2)**	32.1 (28.0–35.7)	31.4 (27.0–35.3)**
Maternal weight (kg)	77.1 (69.0–88.0)	72.0 (64.0–81.3)**	80.0 (71.6–91.0)	73.9 (65.7–84.0)**
Maternal height (cm)	165 (160–169)	163 (158–167)**	165 (161–170)	163 (158–167)**
Ethnicity				
White	13 652 (71.8)	1564 (58.5)**	72 945 (76.4)	7900 (63.6)**
Black	3823 (20.1)	755 (28.3)**	13 213 (13.8)	2441 (19.7)**
South Asian	728 (3.8)	206 (7.7)**	4635 (4.9)	1322 (10.6)**
East Asian	386 (2.0)	64 (2.4)	1812 (1.9)	297 (2.4)**
Mixed	415 (2.2)	83 (3.1)*	2850 (3.0)	460 (3.7)**
Cigarette smoker	1832 (9.6)	514 (19.2)**	5381 (5.6)	1489 (12.0)**
Mode of conception				
Natural	18 465 (97.2)	2598 (97.2)	91 399 (95.8)	11 877 (95.6)
Ovulation drugs	161 (0.8)	23 (0.9)	509 (0.5)	77 (0.6)
*In‐vitro* fertilization	378 (2.0)	51 (1.9)	3547 (3.7)	466 (3.8)
Medical condition				
Chronic hypertension	235 (1.2)	56 (2.1)**	1002 (1.0)	256 (2.1)**
Diabetes mellitus Type I	70 (0.4)	5 (0.2)	414 (0.4)	23 (0.2)**
Diabetes mellitus Type II	111 (0.6)	23 (0.9)	711 (0.7)	99 (0.8)
Obstetric history				
Nulliparous	8894 (46.8)	1519 (56.8)	42 612 (44.6)	6929 (55.8)
Parous, prior SGA	806 (4.2)	332 (12.4)**	5924 (6.2)	2141 (17.2)**
Parous, no prior SGA	9304 (49.0)	821 (30.7)**	46 919 (49.2)	3350 (27.0)**
GA at screening (weeks)	32.2 (32.0–32.6)	32.2 (32.0–32.6)	36.1 (35.6–36.3)	36.0 (35.6–36.4)**
EFW *Z*‐score	0.13 (−0.50 to 0.78)	−1.02 (−1.60 to −0.44)**	0.13 (−0.43 to 0.68)	−0.98 (−1.88 to 0.40)**
GA at delivery (weeks)	40.1 (39.1–40.9)	39.6 (38.5–40.6)**	39.9 (39.0–40.7)	39.1 (38.0–40.1)**
BW (g)	3480 (3210–3775)	2725 (2500–2880)**	3480 (3215–3770)	2695 (2480–2850)**
BW *Z*‐score	0.10 (−0.49 to 0.71)	−1.74 (−2.17 to −1.48)**	0.13 (−0.44 to 0.74)	−1.72 (−2.11 to −1.46)
BW percentile	54.1 (31.3–76.3)	4.1 (1.5–7.0)**	55.3 (32.9–77.3)	4.3 (1.7–7.1)**

Data are given as median (interquartile range) or *n* (%).

* *P* < 0.05 *vs* birth weight (BW) ≥ 10^th^ percentile.

** *P* < 0.01 *vs* BW ≥ 10^th^ percentile.

EFW, estimated fetal weight; GA, gestational age; SGA, small‐for‐gestational age.

### Performance of screening at 35 + 0 to 36 + 6 *vs* 31 + 0 to 33 + 6 weeks for SGA

**Figure 1:**
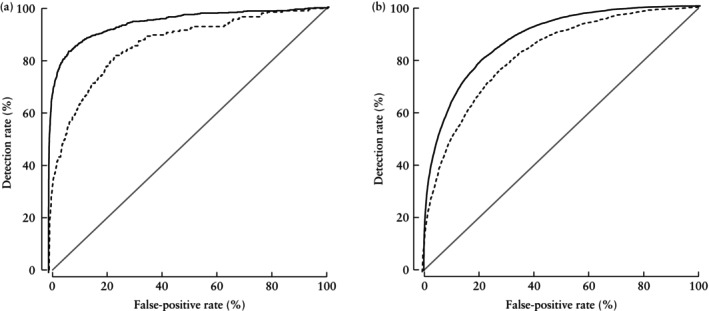
Receiver‐operating‐characteristics curves for screening by estimated fetal weight at 35 + 0 to 36 + 6 weeks' gestation (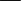) and at 31 + 0 to 33 + 6 weeks (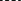) in prediction of small‐for‐gestational‐age neonate with birth weight < 10^th^ percentile delivered at 35 + 0 to 36 + 6 weeks (a) and at any time ≥ 35 + 0 weeks (b).

Table 2 and Figure 1 illustrate the comparison of the predictive performance for delivery of a SGA neonate at 35 + 0 to 36 + 6 weeks' gestation and at any time ≥ 35 + 0 weeks and ≥ 37 + 0 weeks for screening by EFW at 35 + 0 to 36 + 6 *vs* 31 + 0 to 33 + 6 weeks' gestation. Both the AUCs and the DRs at a 10% false‐positive rate for delivery of a SGA neonate with birth weight < 10^th^ or < 3^rd^ percentile were significantly higher for screening at 35 + 0 to 36 + 6 weeks compared with screening at 31 + 0 to 33 + 6 weeks' gestation.

**Table 2:** Comparison of area under receiver‐operating‐characteristics curve (AUC) and detection rate (DR) at 10% false‐positive rate (FPR) in screening for small‐for‐gestational‐age neonate by estimated fetal weight at 31 + 0 to 33 + 6 weeks *vs* 35 + 0 to 36 + 6 weeks

Outcome measure	Screening at 31 + 0 to 33 + 6 weeks	Screening at 35 + 0 to 36 + 6 weeks	*P*
BW < 10^th^ percentile at 35 + 0 to 36 + 6 weeks			
AUC	0.858 (0.830–0.885)	0.944 (0.934–0.954)	< 0.0001
DR at 10% FPR (%)	61.2 (54.4–67.7)	85.7 (83.0–88.1)	
BW < 10^th^ percentile at ≥ 35 + 0 weeks			
AUC	0.817 (0.809–0.826)	0.879 (0.876–0.882)	< 0.0001
DR at 10% FPR (%)	49.4 (47.4–51.2)	63.9 (63.0–64.7)	
BW < 10^th^ percentile at ≥ 37 + 0 weeks			
AUC	0.814 (0.806–0.823)	0.875 (0.872–0.878)	< 0.0001
DR at 10% FPR (%)	48.4 (46.4–50.4)	62.5 (61.6–63.3)	
BW < 3^rd^ percentile at 35 + 0 to 36 + 6 weeks			
AUC	0.901 (0.871–0.932)	0.973 (0.964–0.983)	< 0.0001
DR at 10% FPR (%)	71.9 (63.2–79.5)	94.4 (91.9–96.3)	
BW < 3^rd^ percentile at ≥ 35 + 0 weeks			
AUC	0.852 (0.840–0.863)	0.931 (0.927–0.935)	< 0.0001
DR at 10% FPR (%)	56.7 (53.6–59.7)	79.7 (78.5–80.8)	
BW < 3^rd^ percentile at ≥ 37 + 0 weeks			
AUC	0.865 (0.853–0.876)	0.926 (0.922–0.930)	< 0.0001
DR at 10% FPR (%)	60.7 (57.5–63.9)	78.1 (76.8–79.3)	

Data in parentheses are 95% CI.

BW, birth weight.

### Performance of screening at 35 + 0 to 36 + 6 weeks for SGA at different EFW cut‐offs

The predictive performance for delivery of a SGA neonate with birth weight < 10^th^ or < 3^rd^ percentile of screening by EFW at a series of cut‐offs between the 10^th^ and 50^th^ percentiles at 35 + 0 to 36 + 6 weeks' gestation is shown in Table 3. Screening by EFW < 10^th^ percentile predicted 74% and 86% of neonates with birth weight < 10^th^ percentile and < 3^rd^ percentile, respectively, born within 2 weeks after assessment; the respective values for neonates born at any time after assessment were 43% and 62%.

**Table 3:** Predictive performance for small‐for‐gestational‐age neonate with birth weight < 10^th^ or < 3^rd^ percentile of screening by estimated fetal weight (EFW) below specific percentile cut‐offs at 35 + 0 to 36 + 6 weeks' gestation

		Detection rate			
		Birth weight < 10^th^ percentile		Birth weight < 3^rd^ percentile	
EFW percentile cut‐off	Screen‐positive rate (*n* = 107 875)	Delivery within 2 weeks* (*n* = 3084)	Delivery at any time* (*n* = 12 420)	Delivery within 2 weeks* (*n* = 1691)	Delivery at any time* (*n* = 4724)
< 10^th^	8581 (8.0 (7.7–8.5))	2280 (74 (71–77))	5354 (43 (42–45))	1461 (86 (84–88))	2941 (62 (59–65))
< 15^th^	12 682 (11.8 (11.4–12.3))	2468 (80 (77–83))	6684 (54 (53–57))	1541 (91 (89–93))	3398 (72 (70–75))
< 20^th^	17 057 (15.8 (15.5–16.4))	2617 (85 (82–87))	7798 (63 (62–65))	1581 (93 (92–96))	3821 (81 (77–84))
< 25^th^	21 743 (20.2 (19.9–20.5))	2727 (88 (84–92))	8746 (70 (69–72))	1619 (96 (95–98))	3976 (84 (83–85))
< 30^th^	26 757 (24.8 (24.5–25.1))	2803 (91 (89–93))	9447 (76 (75–79))	1632 (97 (96–98))	4133 (87 (84–90))
< 35^th^	32 170 (29.8 (29.5–30.1))	2875 (93 (92–95))	10 094 (81 (80–83))	1649 (98 (97–99))	4274 (90 (87–93))
< 40^th^	37 538 (34.8 (34.5–35.1))	2932 (95 (94–96))	10 609 (85 (84–86))	1664 (98 (97–99))	4385 (93 (91–95))
< 45^th^	43 139 (40.0 (39.7–40.3))	2974 (96 (95–97))	11 068 (89 (88–90))	1670 (99 (98–100))	4474 (95 (94–96))
< 50^th^	48 857 (45.3 (45.0–45.6))	3002 (97 (96–97))	11 425 (92 (91–93))	1677 (99 (98–100))	4549 (96 (96–97))

Data are given as *n* (% (95% CI)).

* After assessment.

Prediction of ≥ 85% of SGA neonates with birth weight < 10^th^ percentile born at any time after screening at 35 + 0 to 36 + 6 weeks' gestation requires use of EFW < 40^th^ percentile. Screening at this percentile cut‐off predicted 95% and 98% of neonates with birth weight < 10^th^ percentile and < 3^rd^ percentile, respectively, born within 2 weeks after assessment; the respective values for neonates born at any time after assessment were 85% and 93%.

### Performance of screening at 35 + 0 to 36 + 6 weeks for FGR

**Figure 2:**
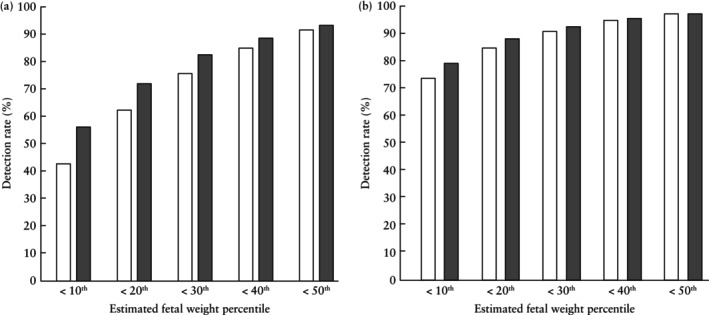
Detection rate for delivery of small‐for‐gestational‐age neonate (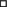) and fetal growth‐restricted neonate (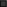) with birth weight < 10^th^ percentile at any time (a) and within 2 weeks (b) after assessment at 35 + 0 to 36 + 6 weeks' gestation.

FGR was diagnosed in 2937 (2.7%) pregnancies undergoing routine screening at 35 + 0 to 36 + 6 weeks' gestation. The predictive performance for SGA and FGR neonates born within 2 weeks and at any time after assessment at 35 + 0 to 36 + 6 weeks' gestation of screening by EFW at a series of cut‐offs between the 10^th^ and 50^th^ percentiles is shown in Table 4 and Figure 2. Screening by EFW < 10^th^ percentile predicted 74% and 79% of SGA and FGR neonates, respectively, born within 2 weeks after assessment; the respective values for neonates born at any time after assessment were 43% and 57%.

**Table 4:** Predictive performance for small‐for‐gestational‐age (SGA) and for fetal growth‐restricted (FGR) neonates with birth weight < 10^th^ percentile of screening by estimated fetal weight (EFW) below specific percentile cut‐offs at 35 + 0 to 36 + 6 weeks' gestation

		Detection rate			
		Delivery within 2 weeks*		Delivery at any time*	
EFW percentile cut‐off	Screen‐positive rate (*n* = 107 875)	SGA (*n* = 3084)	FGR (*n* = 1231)	SGA (*n* = 12 420)	FGR (*n* = 2937)
< 10^th^	8581 (8.0 (7.7–8.5))	2280 (74 (71–77))	978 (79 (76–82))	5354 (43 (42–45))	1663 (57 (53–60))
< 15^th^	12 682 (11.8 (11.4–12.3))	2468 (80 (77–83))	1039 (84 (81–87))	6684 (54 (53–57))	1923 (65 (63–69))
< 20^th^	17 057 (15.8 (15.5–16.4))	2617 (85 (82–87))	1086 (88 (85–91))	7798 (63 (62–65))	2129 (72 (68–77))
< 25^th^	21 743 (20.2 (19.9–20.5))	2727 (88 (84–93))	1121 (91 (88–94))	8746 (70 (69–72))	2306 (79 (77–81))
< 30^th^	26 757 (24.8 (24.5–25.1))	2803 (91 (89–93))	1143 (93 (90–96))	9447 (76 (75–79))	2435 (83 (81–85))
< 35^th^	32 170 (29.8 (29.5–30.1))	2875 (93 (92–95))	1167 (95 (93–97))	10 094 (81 (80–83))	2545 (87 (85–89))
< 40^th^	37 538 (34.8 (34.5–35.1))	2932 (95 (94–96))	1181 (96 (94–98))	10 609 (85 (84–86))	2613 (89 (87–91))
< 45^th^	43 139 (40.0 (39.7–40.3))	2974 (96 (95–97))	1193 (97 (95–99))	11 068 (89 (88–90))	2683 (91 (90–93))
< 50^th^	48 857 (45.3 (45.0–45.6))	3002 (97 (96–97))	1203 (98 (97–99))	11 425 (92 (91–93))	2759 (94 (92–95))

Data are given as *n* (% (95% CI)).

* After assessment.

### Screening by maternal factors plus EFW at 35 + 0 to 36 + 6 weeks for SGA

The adjusted odds ratios for each of the maternal factors in the prediction algorithm are presented in Table 5. The likelihood of SGA decreased with increasing maternal weight and height. The risk was higher in women of black, South Asian or mixed ethnicity compared with white women, as well as in cigarette smokers, those with chronic hypertension and women with a previous pregnancy affected by SGA. The risk was lower in parous women with no history of SGA and in those with diabetes mellitus Type I.

**Table 5:** Fitted regression model including maternal demographic characteristics and medical history for prediction of small‐for‐gestational‐ age (SGA) neonate with birth weight < 10^th^ percentile

	Univariable		Multivariable	
Characteristic	OR (95% CI)	*P*	aOR (95% CI)	*P*
Maternal age − 32 (in years)	0.980 (0.977–0.984)	< 0.001	—	—
Maternal weight − 80 (in kg)	0.969 (0.967–0.970)	< 0.001	0.976 (0.974–0.978)	< 0.001
Maternal height − 165 (in cm)	0.941 (0.938–0.944)	< 0.001	0.966 (0.963–0.969)	< 0.001
Ethnicity				
White	Reference		Reference	
Black	1.706 (1.624–1.792)	< 0.001	2.047 (1.943–2.157)	< 0.001
South Asian	2.634 (2.467–2.812)	< 0.001	1.989 (1.854–2.134)	< 0.001
East Asian	1.513 (1.336–1.715)	< 0.001	1.030 (0.905–1.172)	0.658
Mixed	1.490 (1.347–1.649)	< 0.001	1.434 (1.291–1.592)	< 0.001
Mode of conception				
Natural	Reference		Reference	
*In‐vitro* fertilization	1.030 (0.940–1.129)	0.524	—	—
Cigarette smoker	2.280 (2.146–2.423)	< 0.001	2.654 (2.485–2.833)	< 0.001
Medical disorder				
Chronic hypertension	1.984 (1.727–2.279)	< 0.001	2.505 (2.157–2.910)	< 0.001
Diabetes mellitus Type I	0.426 (0.280–0.649)	< 0.001	0.455 (0.296–0.699)	< 0.001
Diabetes mellitus Type II	1.068 (0.865–1.319)	0.541	1.094 (0.874–1.369)	0.432
Obstetric history				
Nulliparous	Reference		Reference	
Parous, prior SGA	2.223 (2.102–2.350)	< 0.001	1.647 (1.553–1.746)	< 0.001
Parous, no prior SGA	0.439 (0.420–0.459)	< 0.001	0.426 (0.408–0.445)	< 0.001

aOR, adjusted odds ratio; OR, odds ratio.

The maternal factor‐related risk for delivery of a SGA neonate with birth weight < 10^th^ percentile was calculated from the following formula: odds/(1 + odds), where odds = e^Y^ and Y was derived from multivariable logistic regression analysis:

$$\begin{array}{ll} Y & =-2.07540+\left(-0.02429\times \text{maternal weight}\right)\\ & \;+\left(-0.03429\times \text{maternal height}\right)\\ & \;+\left(0.71648\times \text{black ethnicity}\right)\\ & \;+\left(0.68779\times \text{South Asian ethnicity}\right)\\ & \;+\left(0.03643\times \text{mixed ethnicity}\right)\\ & \;+\left(0.97590\times \text{cigarette smoker}\right)\\ & \;+\left(0.91847\times \text{chronic hypertension}\right)\\ & \;+\left(-0.78688\times \text{diabetes mellitus Type}\;I\right)\\ & \;+\left(-0.85317\times \text{parous},\mathrm{no}\;\text{previous}\;\mathrm{SGA}\right)\\ & \;+\left(0.49876\times \text{parous},\text{previous}\;\mathrm{SGA}\right) \end{array}$$

The fitted regression models with maternal factors and EFW *Z*‐score at 35 + 0 to 36 + 6 weeks' gestation for the prediction of a SGA neonate with birth weight < 10^th^ percentile are shown in Table 6. The maternal factor‐derived logit (prior risk) had a DR of 32% and 34% at a false‐positive rate of 10% in screening for delivery at any time after assessment of a SGA neonate born at any time after assessment with birth weight < 10^th^ and < 3^rd^ percentile, respectively (Table 7). At a 10% false‐positive rate, the combination of maternal factors plus EFW *Z*‐score predicted 66% and 75% of SGA neonates born at any time after assessment with birth weight < 10^th^ and < 3^rd^ percentile, respectively; the respective values for EFW < 10^th^ percentile alone were 43% and 62%.

**Table 6:** Fitted regression models with maternal demographic characteristics and medical history (maternal factors) and estimated fetal weight (EFW) *Z*‐score at 35 + 0 to 36 + 6 weeks' gestation for prediction of small‐for‐gestational‐age neonate with birth weight < 10^th^ percentile

Independent variable	Coefficient	SE	OR (95% CI)	*P*
Intercept	−1.33333	0.042	—	—
Maternal factors (logit)	1.60814	0.042	4.99 (4.60–5.42)	< 0.001
EFW *Z*‐score	−1.76668	0.016	0.17 (0.16–0.17)	< 0.001

OR, odds ratio; SE, standard error.

**Table 7:** Predictive performance for delivery of small‐for‐gestational‐age neonate with birth weight (BW) < 10^th^ or < 3^rd^ percentile within 2 weeks and at any time after screening at 35 + 0 to 36 + 6 weeks' gestation

	Delivery within 2 weeks*		Delivery at any time*	
Screening test	AUC	DR at 10% FPR (%)	AUC	DR at 10% FPR (%)
BW < 10^th^ percentile				
Maternal factors	0.720 (0.710–0.731)	31 (29–33)	0.718 (0.713–0.722)	32 (30–34)
EFW < 10^th^ percentile	0.835 (0.825–0.844)	74 (72–76)	0.698 (0.692–0.704)	43 (41–45)
Maternal factors + EFW *Z*‐score	0.935 (0.931–0.940)	80 (78–82)	0.889 (0.886–0.892)	66 (64–68)
BW < 3^rd^ percentile				
Maternal factors	0.712 (0.699–0.725)	30 (27–33)	0.729 (0.722–0.737)	34 (32–36)
EFW < 10^th^ percentile	0.863 (0.853–0.873)	86 (84–88)	0.783 (0.775–0.792)	62 (60–64)
Maternal factors + EFW *Z*‐score	0.937 (0.932–0.943)	82 (78–82)	0.919 (0.915–0.923)	75 (73–77)

Data in parentheses are 95% CI.

* After assessment.

AUC, area under receiver‐operating‐characteristics curve; DR, detection rate; EFW, estimated fetal weight; FPR false‐positive rate.

## DISCUSSION

### Main findings

There are three main findings of this large study of women with a singleton pregnancy undergoing a routine third‐trimester ultrasound scan to predict the delivery of a SGA neonate. First, the performance of this scan is superior to that reported for maternal abdominal palpation and serial measurements of symphysis–fundus height^3^, ^4^. Second, the predictive performance of third‐trimester routine ultrasound for delivery of a SGA neonate is higher if: the scan is carried out at 36 weeks rather than at 32 weeks; the outcome measure is birth weight < 3^rd^ rather than < 10^th^ percentile; the outcome measure is FGR rather than SGA; delivery occurs within 2 weeks after assessment rather than at any time after assessment; and prediction is performed using a model that combines maternal demographic characteristics and elements of medical history with EFW, rather than EFW < 10^th^ percentile alone. Third, at 36 weeks' gestation, detection of ≥ 85% of SGA neonates with birth weight < 10^th^ percentile born at any time after assessment necessitates the use of EFW < 40^th^ percentile.

In a previous study of 124 443 singleton pregnancies examined at 11–13 weeks' gestation, we reported that 85% of SGA neonates are born at ≥ 37 weeks' gestation^11^. In the present study, we found that, for neonates born at ≥ 37 weeks' gestation, sonographic EFW at 36 weeks detects 78% and 63% of SGA neonates with birth weight < 3^rd^ percentile and < 10^th^ percentile, respectively; the respective values for ultrasound examination at 32 weeks are 61% and 48% (Table 2).

The most effective method for predicting the delivery of a SGA neonate is to perform the scan at 36 weeks' gestation and combine maternal risk factors with EFW to define the individual patient‐specific risk. At a 10% false‐positive rate, the DR for delivery of a SGA neonate with birth weight < 3^rd^ percentile and < 10^th^ percentile at any time after assessment is 75% and 66%, respectively, using the combined test, compared to 62% and 43%, respectively, when screening by EFW alone (Table 7).

### Comparison with findings from previous studies

Two studies have demonstrated that a routine late third‐trimester scan is superior to selective ultrasound based on serial symphysis–fundus height measurements in the prediction of a SGA neonate^5^, ^6^. In the first study, 3977 women underwent clinically indicated ultrasonography in the third trimester as per routine clinical care (selective ultrasonography), in addition to research ultrasonography, including fetal biometry at 28 and 36 weeks' gestation, and the research results were not made available to participants or treating clinicians (universal ultrasonography)^5^. The DR for a SGA neonate with birth weight < 10^th^ percentile was 20% for selective ultrasonography and 57% for universal ultrasonography. The second study was a randomized controlled trial of 508 low‐risk pregnant women who underwent either a routine third‐trimester scan at 36 + 0 to 37 + 6 weeks' gestation or selective ultrasound based on serial symphysis–fundus height measurements^6^. The DRs for a SGA neonate with birth weight < 10^th^ and < 3^rd^ percentile were 52.8% and 66.7%, respectively, in the routine‐scan group, compared with only 7.7% and 8.3%, respectively, in the selective‐ultrasound group.

Two randomized trials have compared the performance of ultrasound examination at 36 *vs* 32 weeks' gestation^7^, ^8^. The first trial of 2586 women reported that the predictive performance for a SGA neonate with birth weight < 10^th^ or < 3^rd^ percentile of an ultrasound scan at 36 weeks was superior to that of a scan at 32 weeks; the respective DRs for birth weight < 10^th^ percentile were 39% and 23%, and those for birth weight < 3^rd^ percentile were 61% and 33%^7^. The second trial of 3701 women reported that the predictive performance for a SGA neonate with birth weight < 10^th^ or < 3^rd^ percentile of an ultrasound scan at 35 + 6 weeks was superior to that at 31 + 6 weeks; the respective DRs for birth weight < 10^th^ percentile were 27% and 17%, and those for birth weight < 3^rd^ percentile were 44% and 18%^8^. Our findings corroborate these two trials and are consistent with those of our previous study comparing the predictive performance of fetal biometry for a SGA neonate in 21 989 singleton pregnancies that had undergone routine ultrasound examination at 31 + 0 to 33 + 6 weeks' gestation with that in 45 847 singleton pregnancies that had undergone routine examination at 35 + 0 to 36 + 6 weeks^10^.

Our finding that prediction of a SGA neonate is more effective if EFW is combined with maternal demographic characteristics and elements of medical history, compared with using EFW alone, lends further support to the findings of two previous studies^11^, ^12^.

### Implications for clinical practice

In the proposed pyramid of pregnancy care, all women should be offered ultrasound‐based assessment at 12, 20 and 36 weeks' gestation^20^. An integrated visit at 11–13 weeks' gestation, in which biophysical and biochemical markers are combined with maternal demographic characteristics and medical history, aims to: identify pregnancies at high risk of fetal trisomy^21^, ^22^, ^23^; diagnose major fetal defects and many genetic syndromes^24^, ^25^, ^26^; identify multiple pregnancy, determine chorionicity and predict adverse outcome^27^, ^28^, ^29^; and predict and prevent preterm pre‐eclampsia and early SGA and, through use of aspirin, reduce the prevalence of these complications^30^, ^31^, ^32^, ^33^, ^34^, ^35^, ^36^. The objectives of a visit at around 20 weeks' gestation are to: examine fetal anatomy, growth and placentation; assess the risk for preterm pre‐eclampsia, preterm SGA and placental dysfunction related stillbirth, based on a combination of maternal risk factors, EFW and UtA‐PI, and stratify subsequent pregnancy care accordingly^37^, ^38^, ^39^, ^40^, ^41^, ^42^, ^43^; and measure cervical length to assess the risk of preterm birth, and reduce this risk by treating women with a short cervix with vaginal progesterone^44^, ^45^. A routine 36‐week scan is useful for: prediction of small and large neonates^46^; diagnosis of fetal abnormalities^47^; diagnosis and management of non‐cephalic presentation^48^; assessment of the risk for term pre‐eclampsia and reduction of this risk by timed birth^49^, ^50^, ^51^; and prediction of adverse perinatal outcome^52^.

In the case of SGA, the 36‐week scan targets the group of pregnancies that constitutes 85% of all cases of SGA, namely those born at term. Identification of cases of SGA delivered before 37 weeks requires assessment at 20 weeks and, on the basis of the derived risks, stratification of care for additional ultrasound examinations^39^, ^41^. The high‐risk group could have a scan at 26–28 weeks' gestation and then again at 32 and 36 weeks if not delivered; the moderate‐risk group would be reassessed at 32 and 36 weeks; and the low‐risk group would be reassessed at 36 weeks^39^. Each assessment would then identify a very high‐risk group in need of intensive monitoring, including fetal growth, fetal Doppler profile and fetal heart‐rate patterns, to define the best plan for delivery.

At 36 weeks' gestation, the predictive performance of EFW < 10^th^ percentile for a SGA neonate is modest for those born within 2 weeks after assessment (86% and 74% for neonates with birth weight < 3^rd^ and < 10^th^ percentile, respectively), but poor for those born at any time after assessment (62% and 43%, respectively) (Table 3). In a previous study, we reported that improved screening performance, especially for pregnancies delivering beyond 2 weeks after assessment, is potentially achieved by a new approach for stratifying pregnancies into four management groups based on EFW and Doppler indices^53^. A very small high‐risk group would require monitoring from the time of the initial assessment and up to delivery; an intermediate‐risk group would require reassessment 2 weeks after the initial assessment; a low‐risk group would require reassessment 4 weeks after the initial assessment; and a large very‐low‐risk group would have no further scans.

### Strengths and limitations

The strengths of this third‐trimester screening study for SGA neonates are as follows. First, it was a prospective examination of a large population of pregnant women attending for routine assessment of fetal growth and wellbeing at either 31 + 0 to 33 + 6 or 35 + 0 to 36 + 6 weeks' gestation. Second, trained sonographers carried out fetal biometry according to a standardized protocol, and we applied a widely used model for calculation of EFW^13^, which has been shown to be the most accurate among 70 previously reported models^14^. Third, the FMF fetal and neonatal reference ranges were used, which have a common median^19^. Finally, we used Bayes' theorem to combine the prior risk from maternal demographic characteristics and medical history with EFW to estimate patient‐specific risks and determine the performance of screening for SGA with varying severity delivered at selected intervals after the time of assessment.

A limitation of this study, in relation to the comparison of the predictive performance for a SGA neonate of scanning at 31 + 0 to 33 + 6 weeks *vs* at 35 + 0 to 36 + 6 weeks, is that the study was not randomized. However, the findings are valid because, during the two consecutive periods of study, the characteristics of the populations were similar, the two hospitals were the same and the ultrasonographers carrying out the scans had received the same training and followed the same protocol for conducting the scan. Another limitation is that the results of fetal biometry at the 35 + 0 to 36 + 6‐week scan were made available to the obstetricians caring for the patients. These obstetricians would have taken specific actions, such as further monitoring in the cases of suspected SGA and, consequently, the performance of screening, particularly in pregnancies delivering within 2 weeks after assessment, would be positively biased.

### Conclusions

About 85% of SGA neonates are born at term^11^. Effective screening for late SGA is provided by a combination of maternal factors and fetal biometry at 35 + 0 to 36 + 6 weeks' gestation.

## Data Availability

Research data are not shared.
